# The health, poverty, and financial consequences of a cigarette price increase among 500 million male smokers in 13 middle income countries: compartmental model study

**DOI:** 10.1136/bmj.k1162

**Published:** 2018-04-11

**Authors:** Global Tobacco Economics Consortium

## Abstract

**Objective:**

To examine the impact of a 50% increase in market prices of cigarettes on health, poverty, and financial protection.

**Design:**

Compartmental model study.

**Setting:**

13 middle income countries, totalling two billion men.

**Participants:**

500 million male smokers.

**Main outcome measures:**

Life years gained, averted treatment costs, number of men avoiding catastrophic healthcare expenditures and poverty, and additional tax revenue by income group.

**Results:**

A 50% increase in cigarette prices would lead to about 450 million years of life gained across the 13 countries from smoking cessation, with half of these in China. Across all countries, men in the bottom income group (poorest 20% of the population) would gain 6.7 times more life years than men in the top income group (richest 20% of the population; 155 *v* 23 million). The average life years gained from cessation for each smoker in the bottom income group was 5.1 times that of the top group (1.46 *v* 0.23 years). Of the $157bn (£113bn; €127bn) in averted treatment costs, the bottom income group would avert 4.6 times more costs than the top income group ($46bn *v* $10bn). About 15.5 million men would avoid catastrophic health expenditures in a subset of seven countries without universal health coverage. As result, 8.8 million men, half of them in the bottom income group, would avoid falling below the World Bank definition of extreme poverty. These 8.8 million men constitute 2.4% of people living in extreme poverty in these countries. In contrast, the top income group would pay twice as much as the bottom income group of the $122bn additional tax collected. Overall, the bottom income group would get 31% of the life years saved and 29% each of the averted disease costs and averted catastrophic health expenditures, while paying only 10% of the additional taxes.

**Conclusions:**

Higher prices of cigarettes provide more health and financial gains to the poorest 20% than to the richest 20% of the population. Higher excise taxes support the targets of the sustainable development goals on non-communicable diseases and poverty, and provides financial protection against illness.

## Introduction

On current smoking patterns where large numbers of young adults start smoking but few quit, smoking will be responsible for about one billion deaths in the 21st century.^1^ Most of these will be in low and middle income countries. At the global level, tobacco control relies on the Framework Convention on Tobacco Control^2^ and increasingly on the United Nations 2030 sustainable development goals. The latter include goals to eradicate extreme income poverty, reduce the age standardised death rates from non-communicable diseases by one third, and achieve universal health coverage so as to provide financial risk protection against the impoverishment that arises from illness.^3^ These three goals are interrelated.

Tobacco use is the leading risk factor for non-communicable diseases.^1^ In most countries, smoking prevalence and rates of smoking attributable diseases are highest in lower income stratums.^4^ Smoking accounts for much of the difference in risk of death among men of different social status.^5^ The World Health Organization has estimated that 100 million people fall into poverty (defined by low food expenditure) annually due to out-of-pocket health expenditures,^6^ with much of these expenditures for the treatment of non-communicable diseases.

Progress towards goals for non-communicable diseases is possible only if tobacco cessation rates in most low and middle income countries increase substantially.^1^ ^7^ ^8^ Effective tobacco control could avoid hundreds of millions of premature deaths in the 21st century, and tobacco taxation is the single most effective intervention to increase cessation rates among current smokers and to decrease initiation by young people. The effects of taxes are greatest among young people and people on a low income.^9^ ^10^ However, high excise taxes, at the levels recommended by WHO and the World Bank^11^ remain uncommon in most low and middle income countries.^2^ ^12^

The relation between higher tobacco taxes and poverty levels, impoverishment due to medical treatment costs, and the financial burden of higher taxes in both low and high income groups have been published for China^13^ and Lebanon.^14^ Broad representative assessments across a range of countries have not yet been done. Here, we quantify the likely effect of a 50% cigarette price increase on health, poverty, and financial outcomes in 13 middle income countries with diverse socioeconomic demographic characteristics, tobacco use, and effective universal health coverage.

## Methods

We developed a simple compartmental model to assess the health gains, financial protection, and tax gains for governments from a large increase in the market prices of cigarettes across income groups.^15^ This model was developed by the Disease Control Priorities Project building on an earlier poverty and tobacco taxation analysis by the Asian Development Bank.^13^ ^15^ ^16^ Among the current cohort of smokers in 13 countries, we calculated the cumulative effect of a one-time 50% increase in the market prices of cigarettes on life years gained, treatment costs averted, number of men avoiding catastrophic health expenditures and extreme poverty, and additional tax revenues collected. The supplementary appendix (pages 3, 4, and 13-20) provides the details of the theoretical foundation, data inputs and sources, and statistical procedures.

### Study population

We selected 13 middle income countries in Latin America and Asia, with a total of two billion men. For the compartment model we chose countries based on the prevalence of smoking, population size, and availability of data. Using the World Bank income definitions,^17^ six countries are classified as lower middle income (India, Indonesia, Bangladesh, the Philippines, Vietnam, and Armenia) and seven as upper middle income (China, Mexico, Turkey, Brazil, Colombia, Thailand, and Chile). We focused on male smokers, as they comprised about 90% of all smokers in these 13 countries.^18^ To estimate the number of smokers by five year age group and five income groups (where the bottom income group refers to the poorest 20% of the population and the top group refers to the richest 20% of the population) in each country, we applied the male smoking prevalence by age group from the most recent rounds of the global adult tobacco survey or similar nationally representative surveys (see supplementary appendix pages 13-15) to the UN 2015 population estimates.^19^ Because these surveys do not usually report household income, we used asset index or education as a proxy measure to estimate the smoking prevalence by income group.^20^

### Price effects on smoking

In the main analyses we estimated the cumulative number of smokers who would quit as a consequence of a one-time 50% increase in the retail price of cigarettes. Quitting is a function of price elasticity of demand for cigarettes, age, and income. Studies on cigarette price elasticity (defined by the percentage reduction in cigarette consumption resulting from a specific increase in price) have mostly been done in high income countries but are increasingly available for low and middle income countries.^9^ ^10^ Price elasticity estimates vary widely across countries, time periods, and study design, but reviews of all reliable evidence from both high income countries and low and middle income countries found that elasticity estimates fall within the range of −0.2 to −0.6, or an average of −0.4.^9^ ^10^ The small number of studies in low and middle income countries found smokers to be at least as sensitive (and often more sensitive) to price than smokers in high income countries. A price elasticity of −0.4 implies about a 20% decrease in smoking with a 50% price increase. Of the reduction, roughly half (10%) is attributable to quitting by current smokers and half to fewer cigarettes smoked. Economic theory predicts that those on a low income and young people should be more sensitive to price than others, and this has been well documented in high income countries and in the more limited literature from low and middle income countries.^9^ Price responsiveness in young people and among smokers on a low income is about twice as great as it is in older people and smokers on a high income.^9^ ^21^ The International Agency for Research on Cancer found all 18 price elasticity studies in low and middle income countries reviewed to show a gradient by income or education.^9^

We applied a relative weighted price elasticity matrix by age and income group to all estimates. Hence price elasticity in younger smokers (age 15-24 years) in the bottom income group was −1.27 whereas in smokers aged 25 or more in the top group it was −0.24. We applied the higher price elasticity to future smokers aged less than 15 years who have not yet started to smoke. Sensitivity analyses examined the key outcomes by excluding China and India (as these have more than two thirds of the male smokers of all 13 countries in the study), including the three countries (Chile, Colombia, and Mexico) with notable smoking in females, and testing price increases by 25% and 100% with the above elasticities. We also applied country specific price elasticities (see supplementary appendix pages 3 and 4).

### Price effects on life years gained, disease costs, income poverty, and taxes paid

On the basis of well established effects of quitting we calculated the total life years gained as a result of quitting by age group and income group. We relied on epidemiological studies of populations in high and middle income countries, which document that smoking is responsible for deaths in at least half of the current and future smokers who begin early in adult life and do not quit. Smokers lose an average of 10 years of life compared with otherwise similar non-smokers.^1^ ^22^ ^23^ ^24^ ^25^ ^26^ ^27^ It is reasonable to apply this risk to the 13 countries in the analysis, as most of the current and future smokers studied are aged less than 35 years so many began (or would begin) smoking in early adult life. We applied the benefits of cessation from studies in high-income countries as cessation rates in most low and middle income countries remain low.^18^ ^28^ Various studies in high income countries document that the life years gained from cessation varies by age: approximately 10 years gained for cessation before age 30 years and 9 years, 6 years, and 3 years gained for cessation by ages 30-44 years, 45-64 years, and more than 65 years, respectively.^1^ ^22^ ^23^ ^24^ ^25^ ^26^ We used spline regression to smooth these estimates into five year intervals from 15 to 90 years, assumed similar risk reductions by age across the five income groups, and conservatively excluded any health benefits from fewer cigarettes smoked.

Next we estimated the treatment costs averted due to reduced tobacco attributable death. All costs and prices were in US dollars adjusted for purchasing power parity and expressed in inflation adjusted terms for 2015.^17^ We apportioned the calculated reductions in deaths from the above procedure across four main causes of smoking attributable mortality: chronic obstructive pulmonary disease, stroke, heart disease, and cancers (ignoring tuberculosis). We used global burden of disease estimates of the mortality proportions for these four diseases,^29^ validated with the local epidemiological evidence if available (see supplementary appendix pages 3 and 4).

We derived the annual treatment cost for each country for these four conditions for 2015 (adjusted for purchasing power parity)^17^ from peer reviewed studies or country reports (see supplementary appendix pages 3 and 4). The treatment cost averted was a function of the reduced number of cause specific deaths attributable to tobacco. We quantified the number of men avoiding catastrophic healthcare expenditures using the WHO definition (out-of-pocket costs >10% of an individual’s yearly income)^6^ and extreme poverty as when out-of-pocket costs reduce daily income below the World Bank definition (<$1.90/day).^17^ Because the comparable average individual’s yearly income within each income group were not readily available for all 13 countries, we created a probability distribution of catastrophic expenditures and extreme poverty from an income distribution function for each country based on the Gini coefficient and average per capita household income (see supplementary appendix pages 3 and 4).

Finally, to estimate the value of taxes gained from additional tax revenues from cigarette price increases, we used WHO estimates of country specific data on price per pack of cigarettes ($ purchasing power parity), tobacco tax incidence as a percentage of final price, and average cigarette sticks consumed by smokers each day across income groups.^2^ We used STATA version 13.0 for the analyses. 

### Patient involvement

No patients were involved in setting the research question or the outcome measures nor were they involved in developing plans for implementation of the study. No patients were asked to advise on interpretation or writing up of results.

## Results

We studied 490 million male cigarette smokers in 13 middle income countries (table 1); 291 million were in China and 199 million in the remaining countries. Smoking prevalence varied considerably across countries, as did the number of cigarettes smoked per day. In some countries, such as Indonesia, smoking prevalence was noticeably lower in higher income groups, whereas in Bangladesh and India, cigarette smoking prevalence was similar across income groups. The price (all in $ purchasing power parity) per pack of the most commonly smoked cigarettes varied from $2.20 in Colombia to $10.30 in Turkey. The absolute increase in the median excise tax needed to achieve a 50% price increase was $1.70, ranging from $1.10 in Colombia and the Philippines to $5.10 in Turkey. The median of $1.70 would correspond to an approximate doubling of the excise tax rate, with the assumption that entirety of the excise tax increase is passed on to smokers.

**Table 1:** Key study indicators

Indicators	Lower middle income countries*							Upper middle income countries*						
	India	Indonesia	Bangladesh	Philippines	Vietnam	Armenia		China	Mexico	Turkey	Brazil	Colombia	Thailand	Chile†
Population (2015; in millions)	1311	258	161	101	93	2.9		1376	127	79	208	48	68	18
Male population (2015; in millions)	679	130	81	51	46	1		709	63	39	102	24	34	9
No of people on low income at $1.90/day (2011; $ PPP; in millions)	268	21	28	13	3	0		25	4	0.3	8	3	0.03	1
Total health expenditure as % of GDP	5	3	3	5	7	4		6	6	5	8	7	4	8
Public expenditure on health as % of GDP	1	1	1	2	4	2		3	3	4	4	5	3	4
Out-of-pocket expenditure as % of total health expenditure	62	47	67	54	37	54		32	44	18	25	15	12	32
% of population covered by public financing scheme‡	14	55	26	88	60	28		97	89^§^	85	100	91	98	90
Proportion of costs paid by public financing	40	70	36	41	60	100		26	82^§^	98	81	100	99	90
Male smoking prevalence (15-74 years old)¶	10	58	28	39	46	53		52	21	39	23	18	45	48
Average sticks/day per current smoker	4	12	8	9	11	24		14	10	18	11	8	9	13
No of male cigarette smokers (in millions)	46	53	25	16	15	1		291	10	12	16	3	12	3
Price per pack of cigarettes (2016; in $ PPP)	9.2	5.2	3.4	2.3	2.6	3.1		2.8	5.7	10.3	3.2	2.2	7.1	5.8
Excise tax increase needed for a 50% increase in price (2016; in $ PPP)	4.6	2.6	1.7	1.1	1.3	1.5		1.4	2.9	5.1	1.6	1.1	3.5	2.9
Share of tax to retail price (%)	43.1	57.4	77.0	62.6	35.7	35.0		50.8	67.0	82.1	67.9	49.5	73.5	64.9
% increase in tax rate from baseline tax rate	232	174	130	160	280	286		197	149	122	147	202	136	154
Price per pack after 50% price increase	14	8	5	3	4	5		4	9	15	5	3	11	9

$ 1.00 (£0.72; €0.81).

PPP=purchasing power parity; GDP=gross domestic product.

* World Bank definition.

† World Bank classifies Chile as a high income country, but for present analyses Chile was considered as a middle income country, given that the average household income for Chileans is more or less similar to that of other upper middle income countries such as Brazil

‡ Only public financing schemes considered but mandatory private schemes (eg, ISAPREs for Chile) included. For other countries, private insurance was excluded as it covers only a small portion of the population and is not mandatory.

§ In Mexico, although the universal health coverage rate as well as financial protection provided by Seguro Popular for the first and second groups of income is 100%, the policy only covers chronic obstructive pulmonary disease among tobacco related conditions, whereas the coverage rate for the remaining groups is 82% and financial protection is 70%, and all diseases are covered by health insurance.

¶ Estimates only include cigarettes but exclude bidis mostly used in India and Bangladesh.

The number of male smokers before the price increase was greater in the bottom income group (106 million, or 20%, range 14-27%) than in the top income group (82 million or 17%, range 9-24%); a ratio of 1.3:1 (table 2). A 50% price increase would result in about 67 million men quitting smoking, with the bottom income group having 7.7 times as many quitters as the top income group (23 million *v* 3 million). Cessation would result in about 449 million years of life gained, about half of which would be in China (241 million). Across the 13 countries, the bottom income group would gain 6.7 times more life years than the top income group (155 million *v* 23 million). The average life years gained for each smoker from cessation in the bottom income group was 5.1 times that of the top income group (1.46 *v* 0.23 years). The average life years gained for each smoker would be greatest in young people. At ages 25-29 years, the 50% higher price would lead to 1.4 life years gained for each smoker in the bottom income group compared with 0.3 in the top income group. At ages 60-64 years, the comparable results would be 0.6 and 0.2 life years gained for the bottom and top income groups, respectively (see supplementary appendix page 5).

**Table 2:** Cumulative impact of a 50% cigarette price increase on health and financing outcomes

Variables by income groups	Lower middle income countries*							Upper middle income countries*							Min-Max (%)	Median (%)
	India	Indonesia	Bangladesh	Philippines	Vietnam	Armenia		China	Mexico	Turkey	Brazil	Colombia	Thailand	Chile		
**No of male smokers aged ≥15 years before 50% price increase (in millions)**																
First (bottom 20%)	7.3	13.6	3.0	3.0	3.7	0.1		63.9	1.6	1.8	4.2	0.6	2.8	0.5	14-27	19
Second	10.2	12.0	3.3	2.8	3.3	0.1		68.5	2.0	2.4	3.6	0.6	3.3	0.6	18-27	22
Third	9.5	9.8	3.1	2.6	2.6	0.1		63.1	1.8	2.9	2.9	0.7	2.7	0.7	18-25	21
Fourth	9.1	9.7	3.8	2.5	2.6	0.1		47.7	2.0	2.6	3.1	0.6	2.3	0.7	16-23	19
Group (top 20%)	10.0	7.7	3.0	2.2	2.4	0.1		47.7	2.1	1.9	2.0	0.6	1.0	0.8	8-24	17
Total=490	46.1	52.9	16.2	13.2	14.6	0.6		290.9	9.5	11.6	15.9	3.1	12.0	3.2		
First: group ratio	0.7	1.8	1.0	1.3	1.5	1.2		1.3	0.8	0.9	2.1	0.9	2.7	0.6		
**Total life years gained (in millions)**																
First (bottom 20%)	12.3	22.5	5.4	5.3	5.6	0.1		83.6	3.7	3.3	6.5	0.9	4.5	0.8	26-40	31
Second	13.7	15.8	4.8	4.0	4.1	0.1		71.6	3.8	3.4	4.5	0.8	4.2	0.8	26-32	28
Third	9.4	9.7	3.3	2.8	2.4	0.1		49.2	2.5	3.1	2.7	0.7	2.6	0.7	17-25	20
Fourth	6.0	6.3	2.7	1.8	1.5	0.1		24.6	1.9	1.8	1.8	0.4	1.5	0.5	8-16	12
Group (top 20%)	3.2	2.5	1.1	0.8	0.7	<0.1		12.0	0.9	0.7	0.6	0.2	0.3	0.3	2-8	5
Total=449	44.7	56.8	17.2	14.7	14.3	0.5		241	12.8	12.2	16.1	3.0	13.0	3.1		
First: group ratio	3.8	9.1	5.1	6.9	7.9	6.1		7.0	4.0	4.9	11.0	4.6	14.0	3.1		
**Disease cost averted (adjusted for $ PPP; in millions)**																
First (bottom 20%)	815	4120	81	647	296	16		33400	2170	445	1850	363	878	457	16-34	29
Second	1040	3220	132	538	233	17		35500	2260	566	1720	357	836	494	24-32	27
Third	773	2770	97	405	199	16		24900	1980	524	1170	264	507	436	19-26	22
Fourth	547	2190	136	255	118	10		13400	1600	322	874	168	290	373	11-27	15
Group (top 20%)	313	1050	61	119	73	4		6980	818	132	295	93	64	224	2-12	7
Total=157 002	3488	13 350	507	1964	919	63		114180	8828	1989	5909	1245	2575	1984		
First: group ratio	2.6	3.9	1.3	5.4	4	3.7		4.8	2.7	3.4	6.3	3.9	13.7	2		
**Additional tax revenues (adjusted for $ PPP; in billions)**																
First (bottom 20%)	0.9	2.1	0.2	0.2	0.5	<0.1		9.5	0.3	0.6	0.2	<0.1	0.4	0.1	5-22	10
Second	1.6	2.6	0.4	0.2	0.4	0.1		14.2	0.5	1.6	0.5	0.1	0.8	0.2	13-21	17
Third	1.9	3.4	0.5	0.3	0.4	0.1		14.9	0.4	2.8	0.8	0.1	0.8	0.2	16-26	21
Fourth	2.5	4.8	0.8	0.4	0.5	0.1		12.7	0.8	3.2	0.8	0.1	1	0.3	19-30	26
Group (top 20%)	3.5	3.4	0.8	0.3	0.5	0.1		15	0.9	2.9	0.7	0.1	0.7	0.4	19-35	26
Total=122	10.4	16.4	2.6	1.5	2.4	0.3		66.3	2.9	11.1	3.1	0.4	3.6	1.3		
First: group ratio	0.3	0.6	0.2	0.6	1.1	0.6		0.6	0.3	0.2	0.3	0.2	0.6	0.2		
% additional tax to GDP	**0.1**	**1.0**	**0.1**	**0.2**	**0.4**	**1.0**		**0.2**	**0.1**	**0.7**	**0.1**	**0.1**	**0.3**	**0.3**	**0.1-1.1**	

* World Bank definition; PPP=purchasing power parity; GDP=gross domestic product.

The proportion of health expenditure borne by public health systems and the co-payment requirements for the four diseases varied across countries. The disease costs (all in $ purchasing power parity) that would be averted to treat the four clusters of tobacco attributable diseases would be about $157bn. These averted costs in the bottom income group ($46bn, median 29%, range 16-34%) would be 4.6 times those in the top income group ($10bn, median 7%, range 2-12%). The increases in excise tax needed to achieve a 50% higher price would generate about $122bn across countries, corresponding to between 0.1% and 1.1% of each current country’s gross domestic product in 2015. In contrast to distribution of the health benefits, the extra tax revenue generated from the top income group ($29bn, median 23%, range 19-35%) would be double that from the bottom income group ($15bn, median 10%, range 5-22%).

**Fig 1:**
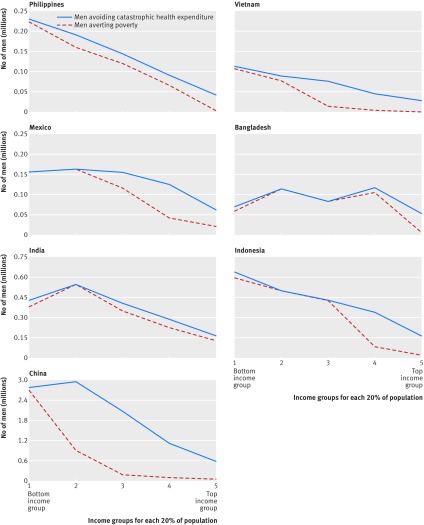
Number of men avoiding catastrophic health expenditures and averting extreme poverty. Catastrophic health expenditure is >10% of individual’s annual income, and extreme poverty is the World Bank’s international poverty line of income of $1.90 (£1.36; €1.54)/day in purchasing power parity

Figure 1 presents the results for poverty and catastrophic expenditures in the six countries with low universal health coverage (India, Indonesia, Bangladesh, the Philippines, Vietnam, and China) and in Mexico, which had high out-of-pocket treatment costs for the four smoking attributable diseases. The 50% higher cigarette price would lead to about 15.5 million men avoiding catastrophic health expenditures and 8.8 million men avoiding extreme poverty, including 4.2 million in the bottom income group (median 37%, range 16-68%; see supplementary appendix page 6) and 2.5 million in the second lowest income group. Nearly all of the extreme poverty avoided was in the bottom income group. The 8.8 million men represent 2.4% of the baseline number of 360 million men and women living in extreme poverty in these seven countries. In most countries, there is an inverse relation between income group and number of people who will avoid catastrophic healthcare expenditures or poverty. In Bangladesh, however, a sizeable number of men who would avoid poverty and catastrophic healthcare expenditures would be from the fourth income group owing to the relatively high prevalence of smoking in this income group.

**Fig 2:**
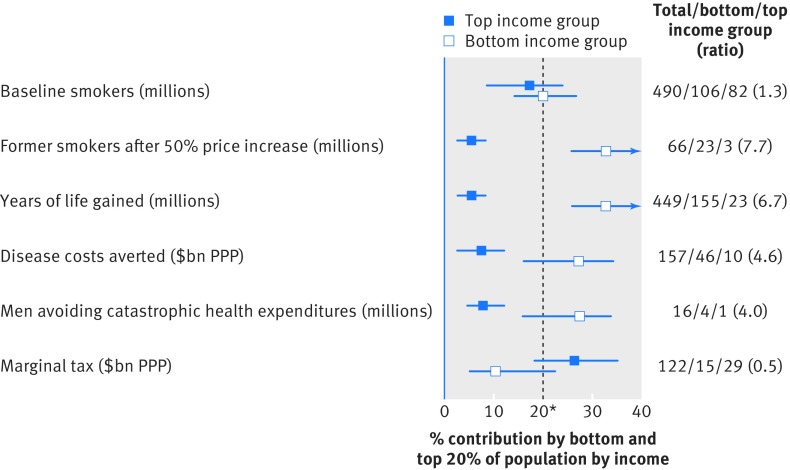
Share of health and financial benefits accruing to bottom and top income groups of population. *Expected value if no differences exist across bottom and top income groups

Figure 2 summarises the differences in the key outcomes for the bottom and top income groups across the 13 countries. Smoking is 1.3 times more common in the bottom income group as the top income group. However, because smokers in the bottom income group are more likely to quit than those in the top income group, the bottom income group would receive a substantially larger share of the health and financial benefits for years of life gained, disease costs averted, and number of people avoiding catastrophic health expenditures. Overall, the bottom income group would get 31% of the life years saved and 29% each of the averted disease costs and averted catastrophic health expenditures but pay only 10% of the additional taxes.

**Fig 3:**
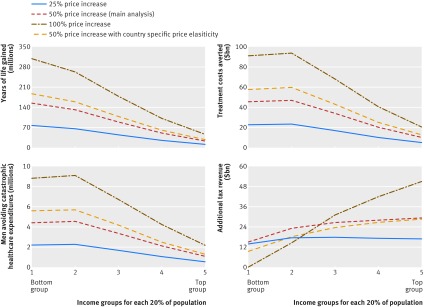
Sensitivity analysis for health and financial outcomes by varying degree of tobacco price increase and using country specific elasticities. $ are in purchasing power parity

Sensitivity analyses yielded similar results. The ratio of catastrophic health expenditures avoided by the bottom versus top income group was 4:1 for all 13 countries and similar (3.5:1) in the 11 countries after exclusion of China and India. This ratio was similar (3.3:1) if we included female smokers from three countries with a relatively high proportion of female smokers to total smokers (Chile at 46% and Colombia and Mexico at 29% each). Use of lower or higher price increases or country specific elasticities showed slightly greater ratios for the bottom versus top income groups (fig 3; see supplementary appendix pages 7-10). The additional tax burden from a 100% price increase would be borne mostly by the top income group.

## Discussion

Across 13 diverse middle income countries, we found that the benefits of tobacco taxation through a 50% price increase favour the bottom income group of the population more strongly for life years saved, out of pocket expenditures from averted tobacco attributable treatment costs, catastrophic health expenditures, and extreme poverty averted. However, a much greater share of the additional tax burden is borne by the top income group. Our results were consistent across countries, despite noticeable differences in smoking prevalence, level of universal health coverage, and poverty levels. Our analysis challenges the conventional view that tobacco taxes are more detrimental to people on low versus high incomes, which is based on the observation that smokers with a low income spend a disproportionately greater share of their income on cigarettes than do high income smokers.^30^

### Relevance of higher taxes to sustainable development goals

Higher tobacco excise taxes support three of the targets of the sustainable development goals on lowering income poverty, reduction of non-communicable diseases, and expanded financial protection against illness. Firstly, in the seven countries with low universal health coverage, practicable increases in tax could avoid about 2.4% of the income poverty by averting out-of-pocket costs for disease treatment. The reduction in poverty is heavily concentrated in the bottom income group but is notable also in the second lowest group, suggesting that higher tobacco taxes help protect those on the borderline of extreme poverty. Higher tobacco excise taxes are a powerful but generally under-appreciated tool to be used by governments to reduce income poverty. Worldwide, some 20 million people could avoid extreme poverty for one year from a 50% higher cigarette price, which compares favourably with 30 million people who have avoided extreme poverty annually in recent years owing to economic growth and other reasons.^31^ Secondly, in these 13 countries alone, some 450 million life years would be saved from higher excise taxes, contributing substantially to the sustainable development goals target of a one third reduction in mortality from non-communicable diseases at ages 30-69 years by 2030.^8^

The relevance of higher tobacco taxes to universal health coverage is more complex. Tobacco taxes can generate substantial revenues, but in most countries they are not enough to meet the financing needs of universal health coverage. Extra tobacco revenue could finance an average of 4% of the recently estimated costs of achieving the health system related sustainable development goals, ranging from 1% in India to 16% in Turkey (see supplementary appendix page 12).^32^ The goals of universal health coverage are not only to improve health but also to reduce poverty through financial risk protection.^6^ Tobacco taxation is an unusually effective way to achieve both. As such, tobacco taxation (within the strategies of the Framework Convention on Tobacco Control) should be a prominent and early intervention in most universal health coverage plans.

WHO observed that between 2012 and 2015 more than 100 countries raised excise taxes on tobacco.^2^ However, few did so at the high levels required to reduce consumption, particularly in many low and middle income countries where rapid income growth has made tobacco relatively more affordable in the past decade.^11^ The median tax increase required to achieve a 50% higher price across the countries was $1.70 for each pack of cigarettes, corresponding to a 100% increase in the excise rate. Although the doubling of excise taxes is not small, the Philippines, Turkey, France, and other countries have adopted comparable or even larger increases.^1^ ^2^ ^33^ The large required increase in excise taxes in some countries mostly reflects the low cost of manufacturing cigarettes. In addition to large tax increases that change consumer behaviour, governments need to pay attention to the structure of the tax and unintended consequences of price differentiation, which can lead to substitution of lesser taxed forms (eg, “cheap, short” cigarettes). In most low and middle income countries—most notably China and Indonesia—the cigarette industry manipulates a wide range of cigarette prices to limit the health effect of any tax increases by encouraging smokers to shift to cheaper brands. The structure in some countries can also create financial incentives for those who engage in tax evasion and avoidance. The World Bank has recently called on governments to implement large, simplified taxes that reduce downward substitution and combat tax avoidance.^11^ Such action requires dispelling common misconceptions about the consequences of higher taxes, most notably on smuggling and tax avoidance (see box 1).

**Box 1 Common misconceptions with supporting evidence about higher tobacco taxation**

- Higher tobacco taxes affect poor people

Smokers on low incomes are more price responsive than wealthier smokers and hence quit (or smoke less) in greater proportions when taxes are higher; thus the health benefits are strongly concentrated in smokers on low incomes^12^ ^13^ ^16^ ^28^
Higher taxes may lead to some smokers switching to cheaper cigarettes. Governments need to pay attention to high specific excise taxes on cigarettes of all lengths to encourage cessation rather than switching (by narrowing the price gap between the most and least expensive cigarettes). A large, simplified tax system can influence consumer behaviour and favour people on low incomes^1^ ^11^ ^28^
In relation to benefits to people on low incomes, spending and taxes both should be considered. Higher taxes enable higher revenue, which might be used to improve health and other social services for people on low incomes^41^

- Higher tobacco taxes lead to more illegal activities such as smuggling and tax avoidance

Legal and illegal tobacco products are not perfect substitutes because there is a high transaction costs involved in consuming illegal products. Higher taxes raise both official and black market retail prices^9^
The main determinant of smuggling is not price but lax enforcement of customs and tolerance for organised criminal smuggling networks^9^ ^41^
Even in the face of moderate smuggling, higher excise tax prices reduce consumption and increase revenue^11^ ^28^ ^41^

- Higher tobacco taxes result in reduced tobacco revenues

The extra revenue per pack of cigarettes outweighs the reduced demand, yielding revenue increases^10^
The World Health Organization estimates that raising tobacco excise tax by Int$1 (about $0.80) in all countries would increase excise revenue by 47%, representing an extra $141bn^10^

- Higher tobacco taxes result in reduced employment

Money not spent on tobacco does not disappear from the economy but is spent on other goods and services that generate employment. This is particularly true for countries where major shares of tobacco leaf or tobacco products are imported
Several reviews of even more extreme curtailing of tobacco use found that increases in tobacco taxes did not lead to net job losses^42^

Smokers, including those on low incomes, who do not quit or substantially reduce their tobacco consumption, will spend more of their income on cigarettes after a tax increase. Those who quit will free up additional income for other expenditures that could enhance their household welfare. Spending on health, education, or other items is reduced in households where men are addicted to tobacco.^34^ ^35^ Although the reductions in smoking related deaths from higher taxes are concentrated in men, the benefits of reductions in catastrophic health expenditures and poverty benefit children, women, and families. Effectively, tobacco taxation enables an income transfer from male smokers to females and other family members. Moreover, tobacco taxes reduce maternal tobacco use, which is an important risk factor for low birth weight and child mortality,^36^ additional targets of the sustainable development goals.

### Limitations of this study

Ideally, direct epidemiological studies in various low and middle income countries would document the hazards of smoking and benefits of cessation by income group. As with any modelling study, ours has certain limitations. Firstly, we used a standard price elasticity of −0.4 across countries. Sensitivity analysis that used country specific elasticities yielded similar poverty effects. Our core premise of a gradient in price elasticity by age and income group is supported by economic theory and most (but not all) studies on price elasticity.^9^ ^21^ Secondly, our model is static, estimating cumulative benefits of a one-time increase and not a longer term reduction in smoking. The ideal would be a dynamic model that incorporates discounting rates and changes in demographic, economic, and healthcare system characteristics over time. However, this is not yet developed. Large, one-time price increases in several countries or states within the United States have been associated with reduced tobacco use.^9^ If also true in low and middle income countries, the one-time price increase would reduce household expenditures on treatment of non-communicable diseases. Thirdly, in theory faster future economic growth among the lower income groups would mean that increases in tobacco taxes may benefit people on low incomes to a lesser extent than we estimate. In reality, the rapid economic growth in the 13 countries continues to be greater in the top income groups (ie, fast developing countries such as China and Brazil have Gini coefficients of 0.46 and 0.53, respectively).^17^ Similarly, a rapid expansion of universal health coverage that reaches people on low incomes would also mean that increases in tobacco taxes are less likely to benefit people on low incomes than we estimate. Unfortunately, the expansion of universal health coverage has generally been slow, and high out-of-pocket costs continue to be the norm in many low and middle income countries.^37^

We also might be underestimating the true benefits of smoking cessation among people on low incomes. We were unable to assess loss of productivity and family earnings related to tobacco use and thereby the greater probability of being pushed into impoverishment. Only about 40% of welfare benefits of disease control broadly arise from averted treatment costs,^38^ with the rest from productivity gains that we did not include. Also, we did not take into account the averted cost related to negative externalities such as second-hand smoking and environmental damage.^39^ We limited our analyses to cigarette smoking. The Indian subcontinent has a sizeable number of bidi (small, locally manufactured cigarettes) users as well as oral tobacco users. In this region, smoking patterns are changing, with cigarettes increasingly substituting bidis, particularly in those on low incomes and young people.^12^, ^40^ Similarly, we also did not account for the modest health benefits of reduced amount of smoking. Finally, our estimates did not take into account the long term signalling effects of higher taxes on individual smoking behaviour. France has halved its daily per capita smoking in only 15 years (the UK took 30 years), in part because its government announced at the outset (in 1992) that excise taxes would rise 5% above inflation every year.^1^ As with mortgages, future rational price expectations can have an additional benefit beyond the initial price increase.

### Implications of this study

Our analyses suggest that large increases in tobacco excise taxation are effective not only at reducing smoking and its consequences on diseases but are also strongly relevant to the UN sustainable development goals for poverty and universal health coverage. Ongoing efforts by countries, the World Bank, WHO, and the Bloomberg Philanthropies and Bill & Melinda Gates Foundation to advance tobacco control can use our findings as new evidence based arguments to accelerate smoking cessation. Modest action by many governments could yield unprecedented health gains and poverty reduction in the 21st century.^41^

**What is already known on this topic**

Higher excise taxes on tobacco are essential to reach the sustainable development goals to reduce mortality from non-communicable diseases by one third by 2030
Low income groups are more responsive to price increases than high income groups
There are few published studies of the distributional impact of higher tobacco taxes on health and financial outcomes

**What this study adds**

Despite differences in socioeconomic class and health finance arrangements a 50% increase in tobacco prices strongly favours those in the bottom income group for life years saved, out-of-pocket expenses from tobacco attributable treatment costs averted, and avoidance of catastrophic health expenditures or poverty
Higher tobacco excise taxes are a powerful but generally underused tool by most governments to reduce expenditures on treatment of diseases that are a major cause of income poverty
In 13 middle income countries studied, around 450 million life years would be saved from higher excise taxes, contributing substantially to the target of the sustainable development goals of a one third reduction in mortality from non-communicable diseases at ages 30-69 by 2030

## Web extra

Extra material supplied by authors

**Supplementary information:** explanation of statistical methodology, input parameters, and results from sensitivity analyses

## Notes

Authors: Sujata Mishra, researcher (Centre for Global Health Research, St Michaels Hospital, Toronto, ON, Canada) and PhD student (Institute of Health Policy, Management and Evaluation, University of Toronto, ON, Canada), Valerie Ulep, researcher (Centre for Global Health Research, St Michael’s Hospital) and PhD candidate (Centre for Health Economics and Policy Analysis, McMaster University, Hamilton, ON, Canada), Patricio Marquez, lead public health specialist (World Bank, Washington, DC, USA), Paul Isenman, consultant, (World Bank), Alan Fuchs, senior health economist (World Bank), Blanca Llorente, technical advisor (Anaas Foundation, Bogota, Colombia), Eduardo Banzon, lead health specialist (Asian Development Bank, Metro Manila, Philippines), Sheila Dutta, senior health specialist (World Bank), Hellen Gelband, senior fellow (Centre for Global Health Research, St Michael’s Hospital), Emmanuel Guindon, assistant professor (Centre for Health Economics and Policy Analysis, McMaster University), Mauricio Hernández-Ávila, professor (Instituto Nacional de Salud Pública, Avenida Universidad, Cuernavaca, Morelos, Mexico), Dean T Jamison, professor (University of California San Francisco Global Health Sciences, San Francisco, CA, USA), Rouselle Lavado, health specialist (Asian Development Bank), Gillian Tarr, postdoctoral associate (University of Calgary, Canada), Iryna Postolovska, young professional (World Bank), Luz Myriam Reynales-Shigematsu, researcher (Instituto Nacional de Salud Pública, Avenida Universidad), Owen Smith, senior health economist (World Bank), Prabhat Jha, professor and director (Centre for Global Heath Research, St Michael’s Hospital)

Contributors: PM and PJ are joint senior authors. SM, VU, and PJ conducted the analyses. PJ, SM, VU, PI, and PM wrote the first draft. PJ conceived the study and is principal investigator of the Global Tobacco Economics Consortium. The coauthors provided substantial contributions to the conception or design of the study or acquisition, analysis, or interpretation of data for the study, and helped with drafting the manuscript or revising it critically for important intellectual content. The coauthors approved this version of the manuscript and agree to be accountable for all aspects of the work in ensuring that questions related to the accuracy or integrity of any part of the work are appropriately investigated and resolved. PJ is guarantor for the study, had full access to all of the data in the study, and takes responsibility for the integrity of the data and the accuracy of the data analysis.

Ethical approval: Not required.

Data sharing The parameters are included in the supplementary appendix, and the STATA codes are available on written request to the corresponding author.

Transparency: The manuscript’s guarantor (PJ) affirms that this manuscript is an honest, accurate, and transparent account of the study being reported; that no important aspects of the study have been omitted; and that any discrepancies from the study as planned (and, if relevant, registered) have been explained.
