# Supplementary material for: The health, poverty, and financial consequences of a cigarette price increase among 500 million male smokers in 13 middle income countries: compartmental model study
Source: BMJ. 2018 Apr 11;361:k1162. doi: 10.1136/bmj.k1162 (PMC5894369; doi:10.1136/bmj.k1162)
Supplement: Supplementary file 1 — Supplementary information: explanation of statistical methodology, input parameters, and results from sensitivity analyses [file miss041510.ww1.pdf]

Supplementary appendix: The health, poverty and financial consequences of a cigarette price increase among 500 million male smokers in 13 middle-income countries: a compartmental model study

Supplementary appendix: The health poverty and financial consequences of a cigarette price increase among 500 million male smokers in 13 middle-income countries

Appendix contents	Page
A. **Appendix Table 1: Input parameters for 13 countries**	3-4
B. **Appendix Figure 1: Life-years gained per smoker by age and five income groups (fifths)**	5
C. **Appendix Table 2. Number of individuals avoiding catastrophic health expenditure and impoverishment**	6
D. **Appendix Table 3a: Sensitivity Analysis- additional life years gained (in millions)**	7
E. **Appendix Table 3b: Sensitivity Analysis- Additional tax revenue (in billions)**	8
F. **Appendix Table 3c: Sensitivity Analysis- Number of treatment cost averted (in billions)**	9
G. **Appendix Table 3d: Sensitivity Analysis- Number of individuals averted from catastrophic expenditures (in millions)**	10
H. **Appendix Table 4: Estimated impact excluding China and India, and including females in Colombia Mexico and Chile**	11
I. **Appendix Table 5: Estimated number of resources to achieve 5% government health expenditure/GDP and SDG**	12
J. **Derivation of outcomes**	13-15
K. **References for the supplementary appendix**	16-20

Appendix Table 1: Input parameters for 13 countries

	Lower middle Income						Upper middle Income							
**Indicators**	**IND**	**IDN**	**BGL**	**PHL**	**VNM**	**ARM**	**CHN**	**MEX**	**TUR**	**BRA**	**COL**	**THA**	**CHL**	**Source**
**Population (males in millions)**														(1)
0-4	65.1	12.7	7.8	5.7	3.8	0.1	44.6	6.0	3.5	7.7	1.9	2.0	0.6	
5-9	66.9	11.8	8.0	5.3	34	0.1	42.4	6.2	3.4	7.9	2.0	2.1	0.6	
10-14	66.9	12.1	8.4	5.1	3.6	0.1	40.4	6.2	3.4	8.9	2.1	2.1	0.6	
15-19	64.9	11.8	8.2	5.1	4.5	0.1	42.0	6.2	3.4	8.9	2.1	2.2	0.7	
20-24	62.1	10.7	7.7	4.8	4.4	0.1	55.9	5.6	3.2	8.4	2.1	2.3	0.7	
25-29	58.7	9.9	7.2	4.2	4.0	0.1	67.0	5.0	3.2	8.8	2.0	2.3	0.7	
30-34	54.1	10.7	6.6	3.7	3.6	0.1	51.1	4.8	3.2	8.9	1.9	2.5	0.7	
35-39	47.2	10.0	5.8	3.3	3.3	0.1	48.8	4.7	3.0	8.1	1.7	2.8	0.6	
40-44	41.8	9.3	5.0	3.0	3.0	0.1	61.0	4.0	2.6	7.0	1.5	2.8	0.6	
45-49	36.5	8.3	4.5	2.7	2.7	0.1	62.7	3.4	2.3	6.4	1.5	2.8	0.6	
50-54	31.9	6.9	3.7	2.3	2.2	0.1	50.6	2.9	2.0	5.9	1.3	2.5	0.6	
55-59	26.9	5.6	2.6	1.9	1.5	0.1	40.1	2.2	1.7	4.8	1.1	2.2	0.5	
60-64	21.7	4.0	1.7	1.4	0.8	0.1	39.2	1.8	1.3	3.8	0.9	1.7	0.4	
65-69	14.2	2.5	1.5	1.0	0.5	<0.1	25.4	1.3	0.9	2.7	0.6	1.2	0.3	
70-74	9.6	1.7	1.1	0.6	0.4	<0.1	16.8	1.1	0.7	1.8	0.4	0.8	0.2	
**Smoking prevalence, by age**														(2–14)
15-19	4%	21%	12%	19%	12%	26%	14%	19%	21%	9%	7%	34%	38%	
20-24	9%	47%	29%	29%	42%	35%	49%	29%	47%	20%	19%	52%	46%	
25-29	9%	54%	34%	39%	45%	43%	53%	27%	54%	18%	26%	48%	51%	
30-34	13%	52%	38%	49%	58%	52%	52%	22%	52%	20%	25%	49%	55%	
35-39	12%	51%	36%	49%	62%	60%	58%	24%	51%	24%	21%	50%	56%	
40-44	12%	50%	33%	48%	56%	66%	68%	19%	50%	24%	17%	50%	55%	
45-49	14%	45%	36%	48%	62%	68%	67%	23%	45%	27%	13%	50%	53%	
50-54	12%	42%	31%	47%	60%	67%	58%	21%	42%	29%	16%	47%	49%	
55-59	10%	32%	26%	45%	64%	64%	58%	17%	32%	27%	17%	44%	44%	
60-64	8%	33%	19%	43%	47%	60%	47%	19%	33%	24%	19%	44%	40%	
65-69	7%	20%	18%	40%	45%	55%	38%	15%	20%	20%	21%	34%	35%	
70-74	6%	16%	22%	36%	34%	51%	21%	10%	16%	16%	21%	34%	31%	
**Smoking prevalence, by five income groups (fifths)**														(2–14)
Q1	8%	72%	26%	32%	58%	49%	59%	21%	32%	31%	16%	48%	30%	
Q2	11%	63%	29%	31%	53%	61%	63%	26%	41%	27%	18%	57%	38%	
Q3	10%	52%	26%	28%	42%	59%	58%	24%	50%	22%	19%	46%	46%	
Q4	10%	51%	33%	27%	40%	49%	44%	27%	45%	22%	17%	40%	48%	
Q5	10%	41%	26%	24%	38%	42%	44%	27%	34%	15%	18%	18%	51%	
**Number of cigarettes consumed daily per person**														(2–14)
Q1	4	18	8	10	14	24	16	13	18	6	6	9	18	
Q2	4	19	8	9	10	24	16	10	19	11	8	9	15	
Q3	4	18	7	10	10	24	14	8	18	14	8	7	11	
Q4	4	17	7	9	10	24	13	9	17	11	8	9	11	
Q5	4	16	8	7	9	24	13	8	16	12	10	10	10	
**Share to the total deaths**														(15)
COPD	23%	9%	31%	10%	11%	7%	19%	8%	15%	2%	19%	83%	14%	
Stroke	18%	50%	16%	35%	47%	24%	39%	12%	24%	5%	22%	37%	34%	
Heart disease	44%	40%	49%	49%	28%	63%	30%	47%	46%	7%	52%	33%	42%	
Lung cancer	15%	2%	5%	6%	13%	6%	12%	33%	15%	1%	7%	16%	10%	
**Annual treatment cost from tobacco attributable diseases (in USD PPP-adjusted)**														(7,8,16–28)
COPD	240	2 977	431	601	400	425	2 256	767	1 604	879	1 289	426	552	

Indicators	IND	IDN	BGL	PHL	VNM	ARM	CHN	MEX	TUR	BRA	COL	THA	CHL	Source
	Lower middle Income						Upper middle Income							
Stroke	895	825	431	1 873	866	350	2 197	3 527	1 850	2 963	1 446	937	4 433	
Heart disease	494	3 935	431	774	1 384	1 724	11 774	4 152	1 537	1 484	968	1 163	3 946	
Lung cancer	895	5 372	644	720	1 319	4 781	14 794	11 811	1 902	2 308	10 240	2 399	21 738	
**Probability of seeking care**														(7,8,25,29–34)
COPD	65%	70%	41%	80%	52%	25%	33%	96%	70%	79%	70%	99%	88%	
Stroke	67%	70%	41%	80%	52%	75%	80%	96%	70%	88%	70%	99%	88%	
Heart disease	70%	70%	41%	80%	52%	75%	81%	96%	70%	87%	70%	99%	88%	
Lung cancer	72%	70%	41%	80%	52%	40%	50%	96%	70%	90%	70%	99%	88%	
**Health utilization (relative)**														(7,8,,19,30,35–42)
Q1	0.8	0.6	0.5	0.8	0.6	0.7	0.79	0.8	0.8	0.7	1.0	1.0	0.9	
Q2	0.9	0.7	0.9	0.9	0.7	0.7	0.98	0.8	1.0	0.9	1.1	1.0	1.0	
Q3	1.0	1.0	1.0	1.0	1.0	1.0	1.00	1.0	1.0	1.0	1.0	1.0	1.0	
Q4	1.1	1.2	1.7	1.0	0.9	1.1	1.08	1.1	1.1	1.1	1.1	1.0	1.2	
Q5	1.2	1.5	2.0	1.1	1.2	1.2	1.15	1.1	1.2	1.1	1.2	1.0	1.4	
**Insurance coverage rate**														(7,8,29,43–53)
	11%	55%	26%	88%	60%	28%	97%	91%	85%	100%	91%	98%	90%	
**Financial support**														(26,43,44,46–49,54–56)
	40%	70%	40%	40%	60%	100%	30%	70%	100%	80%	100%	100%	90%	
**Household income per capita (in USD PPP-adjusted)**														(57–65)
	1 559	1 940	1 437	2 888	2 436	2 888	5 405	4183	10 865	7 511	3 075	7 788	9 419	
**Gini**														
	0.3	0.4	0.3	0.4	0.4	0.3	0.5	0.5	0.4	0.5	0.5	0.4	0.5	(66)
**Individual Income (by five income groups (fifths))**														Authors' calculation
Q1	899	1 008	857	1 393	1 309	1 739	2 435	1 861	5 567	3 017	1 192	4 158	3 886	
Q2	1 243	1 478	1 164	2 125	1 883	2 346	3 866	2 972	8 229	5 100	2 055	6 009	6 467	
Q3	1 501	1 841	1 391	2 711	2 326	2 792	5 027	3 886	10 310	6 881	2 797	7 438	8 654	
Q4	1 791	2 264	1 645	3 401	2 831	3 292	6 423	4 980	12 712	9 042	3 721	9 065	11 293	
Q5	2 352	3 104	2 133	4 795	3 823	4 255	9 260	7 227	17 492	13 550	5 641	12 292	16 813	
**Price elasticity**														(28 67–79)
	-0.35	-0.30	-0.49	-0.87	-0.53	-0.56	-0.54	-0.52	-0.39	-0.38	-0.78	-0.39	-0.21	
**PPP conversion factor**														(80)
	19	4 800	31	20	8 836	202	4	10	2	2	1 292	13	376	

India(IND); Indonesia (IDN); Bangladesh (BGD); Philippines (PHL); Vietnam (VNM); Armenia (ARM): China (CHN); Mexico (MEX); Turkey (TUR); Brazil (BRA); Colombia (COL); Thailand (THA); Chile (CHL)

Note: The population values for Armenia are similar across age-group in this table. However, unrounded-off values were used in the analysis.

Appendix figure 1: Life-years gained per smoker by age and income five income groups (fifths)

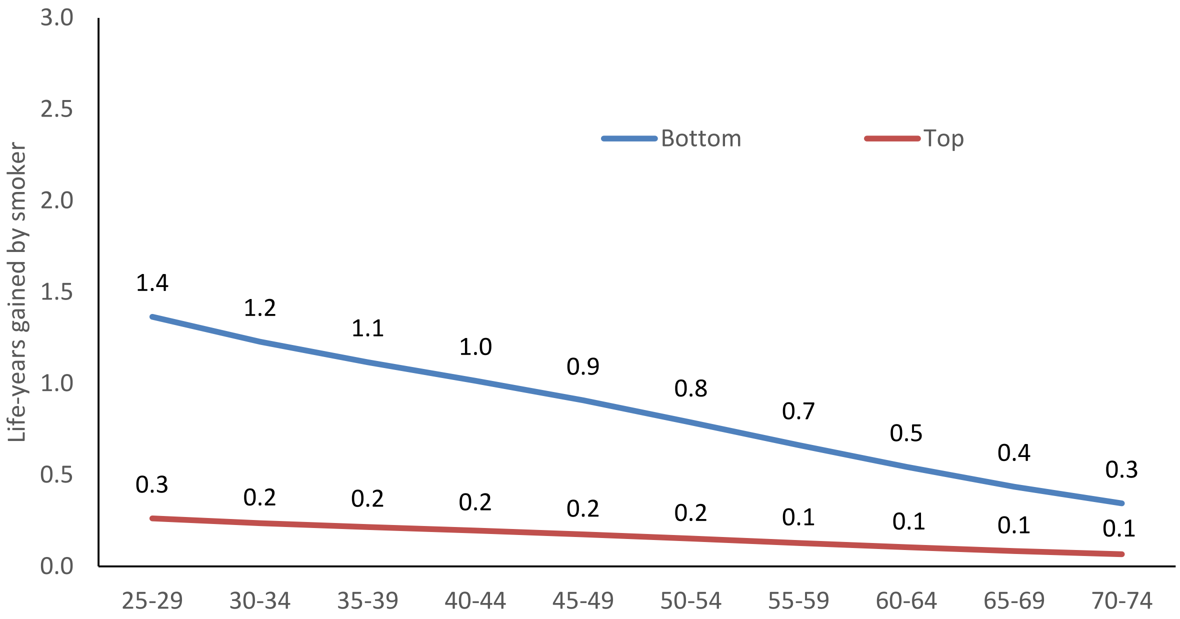

Appendix Table 2. Number of individuals avoiding catastrophic health expenditure and averting poverty

Five income groups (fifths)	Lower middle Income IND	IDN	BGL	PHL	VNM	Upper middle Income CHN	MEX	Range of income group share Min-Max (%)	Median (share)	Mean (share)
**Number of people avoiding catastrophic expenditures from treatment related costs (in millions)**										
Q1 (bottom 20%)	0.43	0.64	0.07	0.23	0.11	2.78	0.16	24-34	29	27
Q2	0.55	0.50	0.11	0.19	0.09	2.95	0.16	24-31	26	27
Q3	0.41	0.43	0.08	0.14	0.08	2.07	0.15	18-23	22	21
Q4	0.29	0.34	0.12	0.09	0.04	1.12	0.13	11-19	16	16
Q5 (top 20%)	0.16	0.16	0.05	0.04	0.03	0.58	0.06	6-10	8	8
Total=15.5	1.83	2.07	0.44	0.70	0.35	9.49	0.66			
Q1/Q5	**2.6**	**3.9**	**1.3**	**5.5**	**4.1**	**4.8**	**2.5**			
**Number of people averting poverty from treatment related costs (in millions)**										
Q1 (bottom 20%)	0.38	0.59	0.06	0.22	0.11	2.69	0.16	16-68	37	38
Q2	0.55	0.50	0.11	0.16	0.08	0.91	0.16	23-37	31	31
Q3	0.35	0.43	0.08	0.12	0.01	0.18	0.12	5-27	21	18
Q4	0.22	0.08	0.11	0.07	<0.01	0.10	0.04	2-12	8	10
Q5 (top 20%)	0.13	0.02	0.01	<0.01	<0.01	0.05	0.02	0-4	1	2
Total=8.8	1.63	1.62	0.37	0.57	0.20	3.93	0.50			
Q1/Q5	**0.2**	**0.4**	**0.2**	**0.4**	**0.5**	**0.7**	**0.3**			

Note: India(IND); Indonesia (IDN); Bangladesh (BGD); Philippines (PHL); Vietnam (VNM); Armenia (ARM): China (CHN); Mexico (MEX); Turkey (TUR); Brazil (BRA); Colombia (COL); Thailand (THA); Chile (CHL). We only include countries with low UHC. We did not include Armenia because of negligible estimates.

Appendix Table 3a: Sensitivity Analysis- additional life years gained (in millions)

	Lower middle Income						Upper middle Income							Range of income group share	Median (share)	Mean (share)
**Five income groups (fifths)**	**IND**	**IDN**	**BGL**	**PHL**	**VNM**	**ARM**	**CHN**	**MEX**	**BRA**	**TUR**	**COL**	**CHL**	**THA**	**Min- Max (%)**		
**25%**																
Q1	6.1	11.2	2.7	2.7	2.8	0.1	41.8	1.9	3.3	1.6	0.5	0.4	2.2	26-40	31	33
Q2	6.8	7.9	2.4	2.0	2.0	0.1	35.8	1.9	2.2	1.7	0.4	0.4	2.1	26-32	28	29
Q3	4.7	4.8	1.6	1.4	1.2	0.1	24.6	1.3	1.3	1.5	0.3	0.4	1.3	17-25	20	20
Q4	3.0	3.2	1.3	0.9	0.8	<0.05	12.3	0.9	0.9	0.9	0.2	0.3	0.7	10-16	12	13
Q5	1.6	1.2	0.5	0.4	0.4	<0.05	6.0	0.5	0.3	0.3	0.1	0.1	0.2	2-8	5	5
Total	**22.3**	**28.3**	**8.6**	**7.3**	**7.1**	**0.2**	**120.5**	**6.4**	**8.0**	**6.1**	**1.5**	**1.5**	**6.5**			
**50%**																
Q1	12.3	22.5	5.4	5.3	5.6	0.1	83.6	3.7	6.5	3.3	0.9	0.8	4.5	26-40	31	33
Q2	13.7	15.8	4.8	4.0	4.1	0.1	71.6	3.8	4.5	3.4	0.8	0.8	4.2	26-32	28	29
Q3	9.4	9.7	3.3	2.8	2.4	0.1	49.2	2.5	2.7	3.1	0.7	0.7	2.6	17-25	20	20
Q4	6.0	6.3	2.7	1.8	1.5	0.1	24.6	1.9	1.8	1.8	0.4	0.5	1.5	10-33	13	17
Q5	3.2	2.5	1.1	0.8	0.7	<0.05	12.0	0.9	0.6	0.7	0.2	0.3	0.3	2-8	5	5
Total	**44.7**	**56.8**	**17.2**	**14.7**	**14.3**	**0.5**	**241.0**	**12.8**	**16.1**	**12.2**	**3.0**	**3.1**	**13.0**			
**100%**																
Q1	24.6	44.9	10.8	10.6	11.2	0.3	167.0	7.4	13.0	6.5	1.8	1.6	9.0	26-40	31	33
Q2	27.3	31.6	9.6	8.1	8.1	0.3	143.0	7.5	8.9	6.8	1.7	1.6	8.4	26-32	28	29
Q3	18.9	19.4	6.6	5.6	4.8	0.2	98.4	5.1	5.4	6.1	1.3	1.4	5.1	17-25	20	20
Q4	12.0	12.6	5.4	3.5	3.1	0.1	49.2	3.8	3.7	3.6	0.8	1.0	2.9	10-16	12	13
Q5	6.4	4.9	2.1	1.5	1.4	<0.05	24.0	1.9	1.2	1.3	0.4	0.5	0.6	2-8	5	5
Total	**89.2**	**113.4**	**34.4**	**29.3**	**28.6**	**1.0**	**481.6**	**25.7**	**32.2**	**24.3**	**6.0**	**6.2**	**26.0**			
**50% (country-specific elasticity)**																
Q1	10.7	16.8	6.6	11.6	7.4	0.2	113.0	4.8	6.1	3.2	1.8	0.4	4.4	26-40	31	33
Q2	11.9	11.9	5.9	8.8	5.4	0.2	96.6	4.9	4.2	3.3	1.6	0.4	4.1	26-32	28	29
Q3	8.2	7.3	4.0	6.1	3.2	0.1	66.4	3.3	2.5	3.0	1.3	0.4	2.5	17-25	20	20
Q4	5.2	4.7	3.3	3.8	2.0	0.1	33.2	2.4	1.7	1.8	0.8	0.3	1.4	10-52	13	18
Q5	2.8	1.8	1.3	1.7	0.9	<0.05	16.2	1.2	0.6	0.6	0.4	0.1	0.3	2-8	5	5
Total	**38.8**	**42.6**	**21.1**	**31.9**	**18.9**	**0.7**	**325.4**	**16.7**	**15.1**	**11.9**	**5.9**	**1.6**	**12.8**			

Note: India(IND); Indonesia (IDN); Bangladesh (BGD); Philippines (PHL); Vietnam VNM); Armenia (ARM): China (CHN); Mexico (MEX); Turkey (TUR); Brazil (BRA); Colombia (COL); Thailand (THA); Chile (CHL). We only include countries with low UHC. We did not include Armenia because of negligible estimates.

Appendix Table 3b: Sensitivity Analysis- Additional tax revenue (in billions)

Five income groups (fifths)	IND	IDN	BGL	PHL	VNM	ARM	CHN	MEX	BRA	TUR	COL	CHL	THA	Range of income group share Min- Max (%)	Median (share)	Mean (share)
	Lower middle Income						Upper middle Income									
**25%**																
Q1	0.7	2.0	0.2	0.2	0.4	<0.05	8.2	0.3	0.2	0.8	<0.05	0.1	0.5	9-27	15	15
Q2	1.2	2.0	0.3	0.2	0.3	<0.05	10.3	0.4	0.4	1.5	<0.05	0.2	0.6	16-24	19	19
Q3	1.2	2.3	0.3	0.2	0.3	<0.05	9.7	0.3	0.6	2.1	0.1	0.2	0.5	15-26	20	21
Q4	1.5	3.0	0.5	0.2	0.3	<0.05	7.6	0.5	0.5	2.2	0.1	0.2	0.7	17-27	24	23
Q5	2.0	2.0	0.5	0.2	0.3	<0.05	8.5	0.5	0.4	1.8	0.1	0.3	0.4	15-31	20	21
Total	**6.6**	**11.3**	**1.9**	**1.0**	**1.6**	**0.2**	**44.2**	**2.1**	**2.2**	**8.4**	**0.3**	**1.0**	**2.7**			
**50%**																
Q1	0.9	2.1	0.2	0.2	0.5	<0.05	9.5	0.3	0.2	0.6	<0.05	0.1	0.4	5-22	10	11
Q2	1.6	2.6	0.4	0.2	0.4	0.1	14.2	0.5	0.5	1.6	0.1	0.2	0.8	13-21	17	17
Q3	1.9	3.4	0.5	0.3	0.4	0.1	14.9	0.4	0.8	2.8	0.1	0.2	0.8	16-26	21	21
Q4	2.5	4.8	0.8	0.4	0.5	0.1	12.7	0.8	0.8	3.2	0.1	0.3	1.0	19-30	27	26
Q5	3.5	3.4	0.8	0.3	0.5	0.1	15.0	0.9	0.7	2.9	0.1	0.4	0.7	19-35	23	26
Total	**10.4**	**16.4**	**2.6**	**1.5**	**2.4**	**0.3**	**66.3**	**2.9**	**3.1**	**11.1**	**0.4**	**1.3**	**3.6**			
**100%**																
Q1	<0.05	<0.05	<0.05	<0.05	0.1	<0.05	<0.05	<0.05	<0.05	<0.05	<0.05	<0.05	<0.05	0-4	0	1
Q2	1.3	1.5	0.1	0.1	0.4	<0.05	10.4	0.1	0.2	0.1	<0.05	<0.05	0.2	0-14	7	7
Q3	2.3	3.9	0.4	0.4	0.6	0.1	18.3	0.4	0.9	2.6	0.1	0.2	0.8	14-25	21	20
Q4	3.8	7.2	1.1	0.5	0.8	0.1	19.5	1.1	1.2	4.5	0.1	0.5	1.5	26-40	34	33
Q5	6.2	6.0	1.4	0.6	0.9	0.1	26.4	1.5	1.3	4.7	0.2	0.8	1.2	31-51	35	38
Total	**13.5**	**18.6**	**3.0**	**1.6**	**2.7**	**0.3**	**74.6**	**3.1**	**3.6**	**11.9**	**0.5**	**1.5**	**3.7**			
**50% (country-specific elasticity)**																
Q1	1.1	3.4	<0.05	<0.05	0.3	<0.05	3.0	<0.05	0.3	0.7	<0.05	0.3	0.4	0-17	8	6
Q2	1.8	3.5	0.2	<0.05	0.3	<0.05	8.6	0.3	0.6	1.7	<0.05	0.4	0.8	0-21	17	14
Q3	2.0	4.0	0.4	<0.05	0.4	<0.05	11.5	0.3	0.8	2.9	<0.05	0.4	0.8	5-26	19	19
Q4	2.6	5.3	0.7	0.2	0.5	0.1	11.1	0.7	0.9	3.3	0.1	0.4	1.0	22-40	27	29
Q5	3.6	3.6	0.8	0.3	0.5	0.1	14.2	0.8	0.7	2.9	0.1	0.5	0.7	18-64	27	32
Total	**11.1**	**19.8**	**2.1**	**0.5**	**1.9**	**0.2**	**48.4**	**2.1**	**3.3**	**11.4**	**0.2**	**2.0**	**3.7**			

Note: India(IND); Indonesia (IDN); Bangladesh (BGD); Philippines (PHL); Vietnam (VNM); Armenia (ARM): China (CHN); Mexico (MEX); Turkey (TUR); Brazil (BRA); Colombia (COL); Thailand (THA); Chile (CHL)

Appendix Table 3c: Sensitivity Analysis- Number of treatment cost averted (in billions)

	Lower middle Income						Upper middle Income									
Five income groups (fifths)	IND	IDN	BGL	PHL	VNM	ARM	CHN	MEX	BRA	TUR	COL	CHL	THA	Range of income group share Min- Max (%)	Median (share)	Mean (share)
**25%**																
Q1	0.4	2.1	<0.05	0.3	0.1	<0.05	16.7	1.1	0.9	0.2	0.2	0.2	0.4	16-34	29	28
Q2	0.5	1.6	0.1	0.3	0.1	<0.05	17.7	1.1	0.9	0.3	0.2	0.2	0.4	24-32	27	27
Q3	0.4	1.4	0.0	0.2	0.1	<0.05	12.4	1.0	0.6	0.3	0.1	0.2	0.3	19-26	22	22
Q4	0.3	1.1	0.1	0.1	0.1	<0.05	6.7	0.8	0.4	0.2	0.1	0.2	0.1	11-27	15	16
Q5	0.2	0.5	0.0	0.1	0.0	<0.05	3.5	0.4	0.1	0.1	0.0	0.1	<0.05	2-12	7	7
Total	**1.7**	**6.7**	**0.3**	**1.0**	**0.5**	**<0.05**	**57.0**	**4.4**	**3.0**	**1.0**	**0.6**	**1.0**	**1.3**			
**50%**																
Q1	0.8	4.1	0.1	0.6	0.3	<0.05	33.4	2.2	1.9	0.4	0.4	0.5	0.9	16-34	29	28
Q2	1.0	3.2	0.1	0.5	0.2	<0.05	35.5	2.3	1.7	0.6	0.4	0.5	0.8	24-32	27	27
Q3	0.8	2.8	0.1	0.4	0.2	<0.05	24.9	2.0	1.2	0.5	0.3	0.4	0.5	19-26	22	22
Q4	0.5	2.2	0.1	0.3	0.1	<0.05	13.4	1.6	0.9	0.3	0.2	0.4	0.3	11-27	15	16
Q5	0.3	1.1	0.1	0.1	0.1	<0.05	7.0	0.8	0.3	0.1	0.1	0.2	0.1	2-12	7	7
Total	**3.5**	**13.4**	**0.5**	**2.0**	**0.9**	**0.1**	**114.2**	**8.8**	**5.9**	**2.0**	**1.2**	**2.0**	**2.6**			
**100%**																
Q1	1.6	8.2	0.2	1.3	0.6	<0.05	66.8	4.3	3.7	0.9	0.7	0.9	1.8	16-34	29	28
Q2	2.1	6.4	0.3	1.1	0.5	<0.05	70.9	4.5	3.4	1.1	0.7	1.0	1.7	24-32	27	27
Q3	1.6	5.5	0.2	0.8	0.4	<0.05	49.8	4.0	2.3	1.1	0.5	0.9	1.0	19-26	22	22
Q4	1.1	4.4	0.3	0.5	0.2	<0.05	26.9	3.2	1.8	0.6	0.3	0.7	0.6	11-27	15	16
Q5	0.6	2.1	0.1	0.2	0.1	<0.05	14.0	1.6	0.6	0.3	0.2	0.4	0.1	2-12	7	7
Total	**7.0**	**26.7**	**1.0**	**3.9**	**1.8**	**0.1**	**228.4**	**17.7**	**11.8**	**4.0**	**2.5**	**4.0**	**5.1**			
**50% (country-specific elasticity)**																
Q1	0.7	3.1	0.1	1.4	0.4	<0.05	45.1	2.8	1.7	0.4	0.7	0.2	0.9	16-34	29	28
Q2	0.9	2.4	0.2	1.2	0.3	<0.05	47.9	2.9	1.6	0.6	0.7	0.3	0.8	24-32	27	27
Q3	0.7	2.1	0.1	0.9	0.3	<0.05	33.6	2.6	1.1	0.5	0.5	0.2	0.5	19-26	22	22
Q4	0.5	1.6	0.2	0.6	0.2	<0.05	18.1	2.1	0.8	0.3	0.3	0.2	0.3	11-27	15	16
Q5	0.3	0.8	0.1	0.3	0.1	<0.05	9.4	1.1	0.3	0.1	0.2	0.1	0.1	2-12	7	7
Total	**3.0**	**10.0**	**0.6**	**4.3**	**1.2**	**0.1**	**154.1**	**11.5**	**5.5**	**1.9**	**2.4**	**1.0**	**2.5**			

Note: India(IND); Indonesia (IDN); Bangladesh (BGD); Philippines (PHL); Vietnam (VNM); Armenia (ARM): China (CHN); Mexico (MEX); Turkey (TUR); Brazil (BRA); Colombia (COL); Thailand (THA); Chile (CHL)

Appendix Table 3d: Sensitivity Analysis- Number of individuals averting catastrophic expenditures from treatment related costs (in millions)

Five income groups (fifths)	Lower middle Income					Upper middle Income		Range of income group share	Median (share)	Mean (share)
	IND	IDN	BGL	PHL	VNM	CHN	MEX	Min- Max (%)		
**25%**										
Q1	0.21	0.32	0.03	0.11	0.06	1.39	0.08	16-33	29	27
Q2	0.27	0.25	0.06	0.10	0.04	1.47	0.08	24-31	26	27
Q3	0.20	0.21	0.04	0.07	0.04	1.03	0.08	19-23	22	21
Q4	0.14	0.17	0.06	0.05	0.02	0.56	0.06	12-27	16	16
Q5	0.08	0.08	0.03	0.02	0.01	0.29	0.03	6-12	8	8
Total	**0.92**	**1.03**	**0.22**	**035**	**0.17**	**4.75**	**0.33**			
**50%**										
Q1	0.43	0.64	0.07	0.23	0.11	2.78	0.16	16-33	29	27
Q2	0.55	0.50	0.11	0.19	0.09	2.95	0.16	24-31	26	27
Q3	0.41	0.43	0.08	0.14	0.08	2.07	0.15	19-23	22	21
Q4	0.29	0.34	0.12	0.09	0.04	1.12	0.13	12-27	16	16
Q5	0.16	0.16	0.05	0.04	0.03	0.58	0.06	6-12	8	8
Total	**1.83**	**2.07**	**0.44**	**0.70**	**0.35**	**9.49**	**0.66**			
**100%**										
Q1	0.86	1.28	0.14	0.46	0.23	5.55	0.31	16-33	29	27
Q2	1.09	1.00	0.23	0.38	0.18	5.90	0.33	24-31	26	27
Q3	0.81	0.86	0.17	0.29	0.15	4.14	0.31	19-23	22	21
Q4	0.57	0.68	0.23	0.18	0.09	2.23	0.25	12-27	16	16
Q5	0.33	0.33	0.11	0.08	0.06	1.16	0.12	6-12	8	8
Total	**3.66**	**4.14**	**0.87**	**1.40**	**0.70**	**18.98**	**1.32**			
**50% country specific elasticity**										
Q1	0.37	0.48	0.15	0.50	0.15	3.75	0.20	23-33	31	29
Q2	0.48	0.37	0.12	0.42	0.12	3.98	0.21	24-31	25	27
Q3	0.35	0.32	0.10	0.31	0.10	2.79	0.20	21-23	22	22
Q4	0.25	0.25	0.06	0.20	0.06	1.51	0.16	12-19	13	14
Q5	0.14	0.12	0.04	0.09	0.04	0.78	0.08	6-9	8	8
Total	**1.59**	**1.55**	**0.46**	**1.52**	**0.46**	**12.81**	**0.86**			

Note: India(IND); Indonesia (IDN); Bangladesh (BGD); Philippines (PHL); Vietnam (VNM); Armenia (ARM): China (CHN); Mexico (MEX); Turkey (TUR); Brazil (BRA); Colombia (COL); Thailand (THA); Chile (CHL)

Appendix Table 4: Estimated impact excluding China and India and including females in Colombia Mexico and Chile

Indicators	13 countries (main analysis)	12 countries (excluding China)	11 countries (excluding China and India)	11 countries (excluding China and India but including females in Chile, Colombia and Mexico)
Number of smokers (in millions)	490	199	153	160
Number of life-years gained (in millions)	449	208	164	171
Disease cost averted (in billion USD) PPP-adjusted	157	43	39	44
Marginal tax gained (in billion USD) PPP-adjusted	122	55	45	47
Number of individuals averting catastrophic expenditure (Q1/Q5)	18.2	18.5	22.2	19.0
Number of individuals averting poverty (Q1/Q5)	4.0	3.2	3.5	3.3

Appendix Table 5: Estimated number of resources to achieve 5% government health expenditure/GDP and SDG

Countries	Government health expenditure		Share of government health expenditure to GDP	Estimated government health expenditure to reach 5% of the current GDP		Deficit per capita (in USD)	Additional revenue		Share of additional revenue to deficit	Share of additional revenue needed to reach 2030 health SDGs
	Total (in million USD)	per capita		Total	per capita		Total (in million USD)	per capita		
India	29 538	23	1.4%	104 442	80	57	3 548	4	7%	1%
Indonesia	9 674	38	1.1%	43 097	167	130	7 779	35	27%	7%
Bangladesh	1 385	9	0.7%	9 754	61	52	901	6	11%	1%
Philippines	4 667	46	1.6%	14 623	145	99	804	6	6%	1%
Vietnam	7 058	77	3.6%	9 680	106	29	983	11	37%	2%
Armenia	210	69	2.0%	526	174	105	119	36	35%	8%
China	321 085	234	2.9%	553 233	403	169	41 065	27	16%	6%
Mexico	44 528	351	3.9%	57 190	450	100	1 427	11	11%	2%
Thailand	12 034	177	3.0%	19 758	291	114	1 233	24	21%	6%
Chile	10 098	563	4.2%	12 040	671	108	924	40	37%	10%
Turkey							3 046	65	NA	16%
Brazil			already attained the target threshold				2 763	14	NA	4%
Colombia							162	4	NA	1%
**Median**	**9886**	**73**	**2.4%**	**17191**	**171**	**103**	**1108**	**18**	**19%**	**4%**

Derivation of outcomes

We estimated the impact of a 50% price increase in cigarette prices on the following health and financial outcomes for each of the 13 countries:

a. Baseline number of male smokers by age and five income groups (fifths)
b. Years of life gained after price intervention
c. Treatment cost averted
d. Individuals averting catastrophic health expenditures and poverty
e. Additional tax revenue

Baseline number of male smokers by age and five income groups (fifths)

Data Sources: (1) 2015 population from UN Population Division; (2) smoking prevalence, by five income groups (fifths) and age-group (5-year) from GATS and similar local surveys.

We defined a current smoker as one who smokes cigarettes either daily or at least once every week. We focused only on manufactured cigarettes and not on bidis, small and locally-grown cigarettes sold commonly in India and Bangladesh.

We used asset index as measure of income. For countries without readily (Available asset index in their respective surveys, we used educational attainment as proxy, and applied the relative prevalence of smoking among illiterate or completion of primary, secondary or high school or college. The following countries have readily: Available asset index: Bangladesh (GATS) Philippines (National Nutrition Survey 2013), Chile Colombia (National Government of the Republic of Colombia. Estudio Nacional de Consumo de Sustancias Psicoactivas 2013-2014), and Mexico (GATS).

Procedure:

In each income group (i) and for each 5-year age group (a), we applied the estimates of smoking prevalence, $Prev_{a,i}$ from the most recent rounds of the Global Adult Tobacco Survey (GATS) or similar nationally representative survey for all $a > 15$. For future smokers i.e. $a < 15$ we assume the same smoking prevalence as for the 15-19 year olds. If P is the population and $P_{i,a}$ is the smoking prevalence of income group i and age group a, then the baseline number, bl of smokers, $Sk_{bl,i,a}$ can be calculated by the following formula:

$$Smk_{\ bl,i,a} = P_{i,a} Prev_{a,i} \qquad \text{(i)}$$

Years of life gained after price intervention

Data Sources: (1) risk-reduction by age-group from Verguet et al; ((81) and (2) model-based estimates from the IHME's Global Burden of Disease.

Procedure*:*

A price increase results in reduction of number of smokers and is subject to the responsiveness of smoker to price change. The price elasticity, ϵ of a smoker in turn is influenced by a and i. As per the literature, the ϵ for cigarettes is about -0.4 meaning a 50% price increase will reduce smoking by about 20%.[82,83] Of this reduction, about half (10%) is attributable to participation elasticity i.e. quitting by current smokers and half to demand elasticity resulting in less amount smoked. Consistent with the published literature showing greater price responsiveness in the young and among the poor[82,83], we doubled the national ϵ among younger smokers (15-24 years old), and also applied this higher price elasticity to future smokers below 15 years old that have not yet started to smoke.[84,85] Similarly, we used a relative weighted price elasticity matrix by income and age drawn from existing studies with the smokers in the bottom fifth (20%) of the population being more price responsive compared to the top fifth. Therefore, the number of quitters is estimated by:

$$Quit_{\ i,a} = Smk_{\ bl,i,a} - Smk_{\ cur,i,a}, \text{ where;}$$

$$Smk_{\ cur,i,a} = Smk_{\ bl,i,a}\left(\frac{1}{2}\epsilon_p \frac{\Delta price}{price} + 1\right) \qquad \text{(ii)}$$

Among persistent smokers, about half of prolonged smokers who do not quit are killed by smoking. This risk is particularly relevant to smokers below age 35 years in LMIC who are likely to have smoked from early in adult life. [86] Here, we conservatively assumed half of current and future smokers would be killed, given that smoking cessation rates in most LMICs are far lower than that in high-income countries. [86,87] Reductions in the excess (all-cause) mortality from smoking are greatest in smokers who quit early in life (and naturally in those who do not start). We applied age-specific benefits of cessation from epidemiological studies in the US and the UK among men and women, [77,88,89] corresponding roughly 97% of smokers avoided excess mortality by quitting by at 15-44 to about 25% avoided excess mortality by quitting by age 65 years. We adopted the risk reduction estimates $RR(a)$ by age group from Verguet et al. Further, we

fitted a cubic spline to derive the age-specific life years gained from smoking cessation for all ages $Y(a)$. [81] To be conservative, we ignored the beneficial effects of reduced smoking amount. We proportioned the reductions in overall mortality across income groups and across four main causes of smoking-related mortality: chronic obstructive pulmonary disease (COPD), stroke, heart disease and tobacco attributable cancers from model-based estimates from the Global Burden of Disease. [15] For China and India, we were able to compare the GBD with direct large epidemiological studies, which yielded generally consistent results for male smoking deaths, but not for women where the GBD estimated wrongly that about 8% of Chinese adult female deaths are due to smoking when the prevalence of adult female smoking is only 2% and even lower in the cohort of women born after 1950. [89] This discrepancy did not, however affect the calculations for males. The total deaths averted are estimated by:

$$D_{averted,i} = (\frac{1}{2}\sum_{a=1}^{18} Quit_{i,a})\ RR(a) \qquad \text{(iii)}$$

Further, the life years gained (LYG) are estimated by:

$$LYG_{i,a} = \left(Quit_{i,a}\right) Y(a) \qquad \text{(iv)}$$

Treatment cost averted
Data Sources: (1) treatment cost, insurance coverage rate, financial support, and healthcare utilization were obtained from peer-reviewed journals and country reports; (2) Purchasing Power Parity (PPP) adjustment factor, and Consumer Price Index were obtained from World Bank

Procedure:
We calculated the treatment cost averted by smokers who quit after price intervention. We obtained local treatment cost estimates, C_d for each of the 4 disease conditions d each country. To equalize the purchasing power of local currencies, we adjusted our cost estimates using a 2015 PPP conversion factor. We estimated the averted total healthcare expenditure (treatment cost), $TC_{averted,i,d}$ conditional to seeking health-care or being ill, HC using the following formula:

$$TC_{averted,i,d} = D_{averted,i,d}\ C_d HC_{i,d} \qquad \text{(v)}$$

We also derived the averted OOP health expenditure, $OOP_{averted,i,d}$ by adjusting the treatment cost with coverage rate of the publicly-funded system, Cov, probability of seeking health-care conditional on being ill, HC, and the percentage of total costs covered by the public healthcare system, $Copay$:

$$OOP_{averted,i,d} = D_{averted,i,d}\ HC_{i,d}\ EC \text{ where, } EC = Cov\ Copay\ C_d \qquad \text{(vi)}$$

Individuals averting catastrophic health expenditures and poverty
Data Sources: (1) Gini Coefficient from the World Bank; (2) average household income capita (2015) were obtained from statistical offices of countries (PPP-adjusted).

Procedure:
Individuals averting catastrophic health expenditures i.e. greater than 10% of their income, attributable to tobacco: We applied the World Bank definition of poverty i.e. earn less than US$ 1.9 /day/capita, World Health Organization's definition of catastrophic health expenditures meaning when out-of-pocket treatment costs exceed 10% of an individual's income for our analysis. We used average household income per capita obtained from statistics offices of respective countries and Gini Coefficient from World Bank to construct gamma distribution of per capita household income.[90] The probability $P_{i,d}$ of individuals falling into poverty or incurring catastrophic health expenditures was derived from this distribution of household income. We estimated the total number of individuals having catastrophic health care expenditures attributed to out-of-pocket cost $C_d EC$ that would be averted by a 50% increase in price by following formula:

$$\sum_d D_{averted,i,d}\ P_{i,d} HC_{i,d}$$

Additional tax revenue
Data Sources: (1) price of most sold brand cigarette, and the share of tax to retail price from the World Health Organization; (2) average number of cigarette of current smokers from GATS.

Procedure:
The tax collected at the baseline is given by the formula:

$$Total\ tax_{bl,q} = Smk\ _{bl,i,a}\ \left(365\frac{Cig_q}{20}\right)TR_{bl}\ \text{ and,} \quad \text{(vii)}$$

$$Total\ tax_{post,q} = Smk\ _{cur,i,a}\ \left(365\frac{Cig_q}{20}\right)TR_{new}, \text{ where;} \quad \text{(viii)}$$

Cig_q is the average number of sticks consumed by smokers in income group q, TR_{bl} is the tax rate per pack of cigarettes at the baseline, and TR_{new} is the new tax rate post price increase. Thus, marginal tax revenues, $MTax_i$ gained is given by:

$$MTax_i = Total\ tax_{post,q} - Total\ tax_{post,q} \quad \text{(ix)}$$

References for the supplementary appendix:

1. World population prospects: the 2015 Revision New York, NY: United Nations, Department of Economic and Social Affairs, Population Division 2015 [Available from: https://esa.un.org/unpd/wpp/] (Accessed June 15, 2017)
2. International Institute for Population Sciences. Global Adult Tobacco Survey: India report. Mumbai, India. Indian Ministry of Health and Family Welfare. 2010.
3. Indonesia Global Adult Tobacco Survey 2011. Ministry of Health, CDC Foundation, U.S. Centre for Disease Control and Prevention, World Health Organization. Jakarta. 2012.
4. Ministry of Health and Family Welfare. Global Adult Tobacco Survey: Bangladesh report. World Health Organization, 2009.
5. Food and Nutrition Research Institute. 8th National Nutrition Survey: report (Philippines). Taguig City. 2014.
6. Ministry of Health Viet Nam, Hanoi Medical University, General Statistics Office of Vietnam, U.S. Centre for Disease Control and Prevention, World Health Organization. Global Adult Tobacco Survey Viet Nam-2010. 2010.
7. Postolovska I, Lavado RF, Tarr G, Verguet S. Estimating the distributional impact of increasing taxes on tobacco products in Armenia: results from an extended cost-effectiveness analysis. Washington DC: The World Bank; 2017.
8. Verguet S, Gauvreau CL, Mishra S, Maclennan M, Murphy SM, Brouwer ED, et al. The consequences of tobacco tax on household health and finances in rich and poor smokers in China: an extended cost-effectiveness analysis. Lancet Glob Heal 2014;3(4):14–8.
9. Instituto Nacional de Salud Publica, U.S. Center for Disease Control and Prevention, CDC Foundation, World Health Organization, Johns Hopkins Bloomberg School of Public Health, International R. Global Adult Tobacco Survey 2015 (Mexico). 2016.
10. Public Health Institution of Turkey, Turkish Statistical Institute, Hacettepe University, CDC Foundation, U.S. Center for Disease Control and Prevention, World Health Organization. Global Adult Tobacco Survey 2012 (Turkey). Ankara: Ministry of Health. 2014.
11. Ministerio de Saude, Instituto de Nacional de Cancer, Instituto Brasileiro de Geografia e Estatistica, U.S. Centers for Disease Control and Prevention B, Pan-American Health Organization, Health. Global Adult Tobacco Survey 2008 (Brazil). 2010.
12. National Government of the Republic of Colombia. Estudio nacional de consumo de sustancias psicoactivas de Colombia 2013. 2014.
13. Ministry of Public Health, World Health Organization, U.S. Centre for Disease Control and Prevention. Global Adult Tobacco Survey 2011 (Thailand). 2011.
14. Ministerio de Salud. Encuesta Nacional de salud Chile 2009-2010. 2010.
15. Institute for Health Metrics and Evaluation (IHME). GBD Compare data visualization. Vol. 2017. Seattle, WA: IHME, University of Washington; 2016. [Available from: http://vizhub.healthdata.org/gbd-compare] (Accessed May 20, 2017)
16. Aras D, Aydoğdu S, Bozkurt E, Cavuşoğlu Y, Eren M, Erol Ç, et al. Cost of heart Failure Management in Turkey: Results of a Delphi Panel. Anatol J Cardio 2016; 16:554–62.
17. Asil T, Celik Y, Sut N, Dogan A, Balci K, Yilmaz A, et al. Cost of acute ischemic and haemorrhagic stroke in Turkey. Clin Neurol Neurosurg 2011;113(2):111–4. [Available from: http://dx.doi.org/10.1016/j.clineuro.2010.09.014] (Accessed May 15, 2017)
18. Tedavi H, Koah E, Maliyeti A. The cost of COPD exacerbations managed in hospital. Turk Toraks Derg 2013; 14:19–23.

19. Pichon-Riviere A, Bardach A, Augustovski F, Alcaraz A, Reynales- Shigematsu LM, Pinto MT, et al. Economic impact of smoking on health systems in Latin America: A study of seven countries and its extrapolation to the regional level. Pan-American Journal of Public Health 2016;40(4):1–9.

20. Ross H, Trung DV, Phu VX. The costs of smoking in Vietnam: the case of inpatient care. Tobacco Control 2007;405–10.

21. Le T, Nguyen T, Nguyen H, Nguyen N. Inpatient treatment cost of stroke: an analysis in Ho Chi Minh City 115 People's Hospital, Vietnam. Value Heal 2016;9(7):649.

22. Sutrisna B, Surtidewi L, Jusuf A, Hudoyo A, Kusmana D, Setianto B, et al. Estimating the annual cost of smoking-related diseases in Indonesia. Media Med 2009;43(18):247–53.

23. Leartsakulpanitch J. The economic burden of smoking-related disease in Thailand: a prevalence-based analysis. J Med Assoc Thai 2007;90(9):1925–9.

24. Kuptniratsaikul V, Kovindha A, Massakulpan P, Kuptniratsaikul PS. Inpatient rehabilitation services for patients after stroke in Thailand: a multi-centre study. J Rehabil Med 2009; 41:684–6.

25. World Health Organization. Impact of tobacco-related illnesses in Bangladesh. 2005.

26. Ulep V, dela Cruz N. Analysis of out-of-pocket expenditures in the Philippines. Philipp J Dev 2013; 1072:93–122.

27. Philippine Health Insurance Corp. Implementing guidelines on medical and procedures case rates. Philippines: Philippine Health Insurance Corporation; 2013. [Available from: https://www.philhealth.gov.ph/circulars/2013/annexes/circ35_2013/] (Accessed June 8, 2017)

28. Fuchs A, Meneses F. Are tobacco taxes really regressive? Evidence from Chile. Washington DC: The World Bank; 2017.

29. Lagomarsino G, Garabrant A, Adyas A, Muga R, Otoo N. Moving towards universal health coverage: health insurance reforms in nine developing countries in Africa and Asia. Lancet 2012;380(9845):933–43. [Available from: http://dx.doi.org/10.1016/S0140-6736(12)61147-7] (Accessed June 15, 2017)

30. Brown C, Pagan J, Rodriguez-Orregia E. The decision-making process of health care utilization in Mexico. Health Policy 2005; 72:81–91.

31. Frenz P, Delgado I, Kaufman JS, Harper S. Achieving effective universal health coverage with equity: evidence from Chile. Health Policy Plan 2014; 717–31.

32. Van Minh H, Xuan Tran B. Assessing the household financial burden associated with the chronic non-communicable diseases in a rural district of Vietnam. Glob Health Action 2012; 5:1–7.

33. Blay SL, Fillenbaum GG, Andreoli SB, Leite F, Blay SL, Fillenbaum GG, et al. Equity of access to outpatient care and hospitalization among older community residents in Brazil. Med Care 2008;46(9):930–7.

34. OECD. OECD Reviews of health care quality: Turkey 2014: raising standards. 2014.

35. Boccolini CS, Roberto P, Souza B De. Inequities in healthcare utilization: results of the Brazilian National Health Survey, 2013. Int J Equity Health 2016;1–8. [Available from: http://dx.doi.org/10.1186/s12939-016-0444-3] (Accessed July 3, 2017)

36. Uddin J, Alam N, Sarma H, Ashique M, Chowdhury H, Alam DS, et al. Consequences of hypertension and chronic obstructive pulmonary disease, healthcare-seeking behaviours of patients, and responses of the health system: a population-based cross-sectional study in Bangladesh. BMC Public Health 2014;1–11.

37. Rokx C, Schieber G, Harimurti P, Tandon A, Somanathan A. Health financing in Indonesia. Washington DC: The World Bank; 2009.

38. Sözmen K, Ünal B. Explaining inequalities in health care utilization among Turkish adults: Findings from Health Survey 2008. Health Policy 2016;120(1):100–10. [Available from: http://dx.doi.org/10.1016/j.healthpol.2015.10.003] (Accessed July 5, 2017)

39. Philippine Statistical Authority, ICF International. 2013 National Demographic and Health Survey Report. Manila, Philippines; 2014.

40. Netithanakul A, Soonthorndhada K. Equity in health care utilization of the elderly: evidence from Kanchanaburi DSS, Thailand. J Popul Soc Stud 2009;18(1):103–22.

41. Núñez A, Chi C. Equity in health care utilization in Chile. Int J Equity Health 2013;1–16.

42. Thi N, Ha H, Berman P, Larsen U. Household utilization and expenditure on private and public health services in Vietnam. Health Policy Plan 2002;17(1):61–70.

43. Amaya-Lara JL. Catastrophic expenditure due to out-of- pocket health payments and its determinants in Colombian households. Int J Equity Health 2016;1–11[Available from: http://dx.doi.org/10.1186/s12939-016-0472-z

44. Boniilla-Chacín ME, Aguilera N. The Mexican social protection system in health. 2013.

45. Bitrán R, Escobar L, Patricia G. After Chile's Health Reform: Increase in coverage and access, decline in hospitalization and death rates. Health Aff 2010; 29(12):2161–2170.

46. Zhang Y, Tang W, Zhang X, Zhang Y, Zhang L. National health insurance development in China from 2004 to 2011: Coverage versus benefits. 2015;1–10.

47. Van Tien T, Thi Phuong H, Mathauer I, Phuong N. A health financing review of Vietnam: with a focus on social health insurance. 2011.

48. Paim J, Travassos C, Almeida C, Bahia L, Macinko J. The Brazilian health system: history, advances and challenges. Lancet 2011; 6736(11).

49. Yardim MS, Cilingiroglu N, Yardim N. Catastrophic Health expenditure and impoverishment in Turkey. Health Policy. Jan;94(1):26-33. [Available from: http://dx.doi.org/10.1016/j.healthpol.2009.08.006] (Accessed July 10, 2017)

50. Mahendradhata YD, Trisnantoro L, Listyadewi S, Harimurti PW, Prawira J. The Republic of Indonesia health system review. Vol. 7. 2017.

51. Philippine Health Insurance Corp. PhilHealth Stats and Charts 2014. 2014.

52. Shijith V, Sekher T. Who gets health insurance coverage in India? New findings from nationwide surveys. In: XXVII IUSSP International Population Conference. 2013. p. 1–26.

53. Mustafa A, Begum T. Universal health coverage assessment People's Republic of Bangladesh. 2014;(December):1–16.

54. Tangcharoensathien V, Patcharanarumol W, Ir P, Aljunid SM, Mukti AG, Akkhavong K, et al. Health-financing reforms in southeast Asia: challenges in. Lancet 2011;377(9768):863–73. [Available from: http://dx.doi.org/10.1016/S0140-6736(10)61890-9] (Accessed May 20, 2017)

55. Huq NM, Al-amin AQ, Howlader SR. Paying out-of-pocket for healthcare in Bangladesh - A burden on poor? Iran J Public Heal 2015;44(7):1024–5.

56. Schrepel C, Tanenbaum E, Paccione GA. How do patients view Chile's AUGE plan? A survey in a rural clinic. Soc Med 2014;8(3):101–12.

57. Instituto Brasileiro de Geografia e Estatistica. Censos Demograficos 2010. [[Available from: ftp://ftp.ibge.gov.br/Censos/Censo_Demografico_2010] (Accessed April 2, 2017)

58. National Bureau of Statistics of China. Household consumption and expenditure 2015. [Available from: http://data.stats.gov.cn/english/easyquery.htm?cn=C01] (Accessed May 2, 2017)

59. National Statistics Office (Thailand). 2015 Household Socio-economic Survey. 2016. [Available from: http://web.nso.go.th/en/survey/house_seco/data/Full_Report2015.pdf] (Accessed May 2, 2017)

60. Philippine Statistical Authority. Income and expenditure. 2016. [Available from: https://psa.gov.ph/survey/annual-poverty-indicator] (Accessed June 18, 2017)

61. General Statistics Office of Vietnam. Monthly average income per capita at current prices by income source and by province. 2010. [Available from: http://www.gso.gov.vn/default_en.aspx?tabid=487&ItemID=4327] (Accessed July 13, 2017)

62. Bangladesh Bureau of Statistics. Bangladesh - Household Income and Expenditure Survey 2010. Dhaka; 2012.

63. Turkish Statistical Institute. Turkish Statistical Institute Income and Living Conditions Survey- 2015. 2016. [Available from: http://www.turkstat.gov.tr/PreHaberBultenleri.do?id=21584] (Accessed July 18, 2017)

64. National Statistics Service of the Republic of Armenia. Household income, expenditure, and basic food consumption 2010-2014. 2015. [Available from: http://www.armstat.am/file/article/6._poverty_2015e_3.pdf] (Accessed June 18, 2017)

65. Instituto Nacional de Estadística y Geografia. 2014 National Survey of Household Expenditure. 2016.

66. World Bank. Gini Index. 2016. [Available from: http://data.worldbank.org/indicator/SI.POV.GINI] (Accessed June 17, 2017)

67. Eozenou P, Fishburn B. Price elasticity estimates of cigarette demand in Vietnam. Florence, Italy; 2001

68. Nargis N, Ruthbah UH, Hussain AKMG, Fong GT, Huq I, Ashiquzzaman SM. The price sensitivity of cigarette consumption in Bangladesh: evidence from the International Tobacco Control (ITC) Bangladesh Wave 1 (2009) and Wave 2 (2010) Surveys. Tob Control. 2014;23 Suppl 1(0 1): i39-47. [Available from: http://www.ncbi.nlm.nih.gov/pubmed/24105828%5Cnhttp://www.pubmedcentral.nih.gov/articlerender.fcgi?artid=PMC4090419] (Accessed June 13, 2017)

69. Adioetomo M, Djutaharta T, Hendratno. Cigarette consumption, taxation, and household income: Indonesia case study. Washington D.C.; 2005.

70. Barber S, Adioetomo M, Ahsan A, Setyonaluri D. Tobacco economics in Indonesia. 2008.

71. John RM. Price elasticity estimates for tobacco products in India. Heal Policy Plan. 2008;23(3):200–9. [Available from: http://www.ncbi.nlm.nih.gov/pubmed/18424474] (Accessed June 28, 2017)

72. Maldonado N, Llorente B, Deaza J. Cigarette taxes and demand in Colombia. Rev Panam Salud Publica.2016;40(4):229–36. [Available from: https://www.ncbi.nlm.nih.gov/pubmed/28001198] (Accessed June 8, 2017)

73. Quimbo SLA, Casorla AA, Miguel-Baquilod M, Medalla FM, Xu X, Chaloupka FJ. The economics of tobacco and tobacco taxation in the Philippines. 2012.

74. Sánchez DD. Economía del control del tabaco en los países del mercosur y estados asociados: Chile. Washington D.C.: Pan American Health Organization; 2006. [Available from: http://iris.paho.org/xmlui/handle/123456789/2811] (Accessed June 18, 2017)

75. Sarntisart I. An economic analysis of tobacco control in Thailand. Health, Nutrition and Population. Washington D.C.: The World Bank; 2003.

76. Jimenez-Ruiz JA, Saenz de Miera B, Reynales-Shigematsu LM, Waters HR, Hernandez-Avila M. The impact of taxation on tobacco consumption in Mexico. Tob Control. 2008;17(2):105–10. [Available from: https://www.ncbi.nlm.nih.gov/pubmed/18285383] (Accessed July 6, 2017)

77. Postolovska I, Lavado R, Tarr G, Verguet S. Estimating distributional impact of increasing tobacco taxes in Armenia: Results from an extended cost-effectiveness analysis. Washington DC: The World Bank; 2017.

78. Hu TW, Mao Z, Shi J, Chen W. The role of taxation in tobacco control and its potential economic impact in China. Tob Control 2010;19(1):58–64. [Available from: https://www.ncbi.nlm.nih.gov/pubmed/20008158] (Accessed July 5, 2017)

79. Yürekli A, Önder Z, Elibol M, Erk N, Cabuk A, Fisunoglu M, et al. The economics of tobacco and tobacco taxation in Turkey. Int Union Against Tuberc Lung Dis 2010

80. World Bank. PPP Conversion Factor. 2016.

81. Verguet S, Tarr G, Gauvreau C, Mishra S, Jha P, Liu L. Distributional benefits of tobacco tax and smoke-free workplaces in China: a modeling study. J Glob Health 2017; in press.

82. Gallet CA, List JA. Cigarette demand: a meta-analysis of elasticities. Heal Econ. 2003;12(10):821–35. [Available from: https://www.ncbi.nlm.nih.gov/pubmed/14508867] (Accessed July 8, 2017)

83. IARC. Effectiveness of tax and price policies for tobacco control: IARC handbook of cancer prevention. Vol. 14. Lyon, France: International Agency for Research on Cancer; 2011.

84. Pirie K, Peto R, Reeves GK, Green J, Beral V, Million Women Study C. The 21st century hazards of smoking and benefits of stopping: a prospective study of one million women in the UK. Lancet 2013;381(9861):133-41.

85. Jha P, Ramasundarahettige C, Landsman V, Rostron B, Thun M, Anderson RN, et al. 21st-century hazards of smoking and benefits of cessation in the United States. N Engl J Med 2013;368(4):341-50.

86. Doll R, Peto R, Boreham J, Sutherland I. Mortality from cancer in relation to smoking: 50 years of observations on British doctors. Br J Cancer 2005;92(3):426–9. [Available from: https://www.ncbi.nlm.nih.gov/pubmed/15668706] (Accessed July 6, 2017)

87. Peto R, Lopez AD, Boreham J, Thun M, Heath Jr. C. Mortality from tobacco in developed countries: indirect estimation from national vital statistics. Lancet 1992;339(8804):1268–78. [Available from: https://www.ncbi.nlm.nih.gov/pubmed/1349675] (Accessed July 6, 2017)

88. Jha P, Joseph RA, Li D, Gauvreau C, Anderson I, Moser P, et al. Tobacco Taxes: A win-win measure for fiscal space and health. Mandaluyong City, Philippines: Asian Development Bank; 2012.

89. Chen Z, Peto R, Zhou M, Iona A, Smith M, Yang L, et al. Contrasting male and female trends in tobacco-attributed mortality in China: evidence from successive nationwide prospective cohort studies. Lancet 2015;386(10002):1447–56. [Available from: https://www.ncbi.nlm.nih.gov/pubmed/26466050] (Accessed July 8, 2017)

90. Kemp-Benedict E. Income distribution and poverty methods for using available data in global analysis. 2001.
